# Retraction: Evaluation of periimplant bone neoformation using different scanning electron microscope methods for measuring BIC. A dog study

**DOI:** 10.4317/jced.56034

**Published:** 2019-05-01

**Authors:** José-Luis Calvo-Guirado, Antonio Aguilar-Salvatierra, Javier Guardia, Rafael Delgado-Ruiz, María-Piedad Ramírez-Fernández, Cristina Pérez-Sánchez, Gerardo Gómez-Moreno

**Affiliations:** 1 Department of Implant Dentistry, School of Medicine and Dentistry, University of Murcia, Spain; 2 Department of Pharmacological Interactions in Dentistry, Dental School, University of Granada, Spain; 3 Master of Implant Dentistry. Faculty of Medicine and Dentistry, University of Murcia

## Abstract

In relation to the article of the Journal of Clinical and Experimental Dentistry “Calvo-Guirado JL, Aguilar-Salvatierra A, Guardia J, Delgado-Ruiz R, Ramírez-Fernández MP, Pérez-Sánchez C, Gómez-Moreno G. Evaluation of periimplant bone neoformation using different scanning electron microscope methods for measuring BIC. A dog study. J Clin Exp Dent. 2012 Feb 1;4(1):e8-e13”, the authors have used three figures that are the same as those published in three different publications (J Pineal Res 2010; COIR 2010; COIR 2012). The copyright of the mentioned publications was consequently not respected. Retraction of the article is therefore decided.

